# Beyond Tumor Mutation Burden: Tumor Neoantigen Burden as a Biomarker for Immunotherapy and Other Types of Therapy

**DOI:** 10.3389/fonc.2021.672677

**Published:** 2021-04-29

**Authors:** Peipei Wang, Yueyun Chen, Chun Wang

**Affiliations:** ^1^ Department of Biotherapy, Cancer Center, West China Hospital, State Key Laboratory of Biotherapy, Sichuan University, Chengdu, China; ^2^ Department of Endocrinology and Metabolism, West China Hospital, Sichuan University, Chengdu, China

**Keywords:** tumor neoantigen burden, biomarker, immunotherapy, immune response, tumor mutation burden

## Abstract

Immunotherapy has significantly improved the clinical outcome of patients with cancer. However, the immune response rate varies greatly, possibly due to lack of effective biomarkers that can be used to distinguish responders from non-responders. Recently, clinical studies have associated high tumor neoantigen burden (TNB) with improved outcomes in patients treated with immunotherapy. Therefore, TNB has emerged as a biomarker for immunotherapy and other types of therapy. In the present review, the potential application of TNB as a biomarker was evaluated. The methods of neoantigen prediction were summarized and the mechanisms involved in TNB were investigated. The impact of high TNB and increased number of infiltrating immune cells on the efficacy of immunotherapy was also addressed. Finally, the future challenges of TNB were discussed.

## Introduction

Tumor immunotherapy aims to control tumor development by activating the immune system to attack tumor cells. By selecting appropriate antigens, notably neoantigens produced by tumor-specific mutations, an effective tumor-specific immune response can be mounted, and immune tolerance can be minimized (1). Non-synonymous somatic mutations will produce altered peptides, among which, some are processed and presented by the major histocompatibility complex (MHC) in order to generate neoantigens. These molecules are the key factors required for successful immunotherapy, including immune checkpoint inhibitors (ICIs), personalized tumor vaccines and adoptive T cell transfer immunotherapy (2–4). These strategies have shown promise in the treatment of solid tumors (5–7).

A higher number of DNA mutations are associated with higher number of candidate peptides, and results in an increased probability of successfully presented neoantigens (8). The response to immunotherapy correlates with tumor mutation burden (TMB) and mainly with the number of mutations in the coding region of the genome (exome) of the tumor cells. It is usually reported as the number of mutations present in a megabase of the genomic region by whole-exome sequencing or large-scale next-generation sequencing (9–12). Similarly, the tumor neoantigen burden (TNB) is defined by the number of neoantigens per megabase in the genome region (13, 14). Notably, TMB has become a biomarker for immunotherapy, assuming that higher TMB will increase the probability of tumor neoantigens and specific T-cell responses (15).

However, the role of TMB in immunotherapy remains controversial (16–18), since not all mutations produce neoantigens. Only a limited number of mutations can be properly processed, presented on the surface of the MHC complex and recognized by T cells (19). The TMB noted in pediatric tumors is considerably low (20). However, in certain tumors, such as pediatric medulloblastoma or acute lymphoblastic leukemia, which exhibit minimal mutational burden, a strong anti-tumor immune response can be induced by high-quality neoantigens (21, 22).

TMB generates neoantigens and causes tumor immunogenicity. This biomarker can be used as a valuable estimate of TNB to a certain extent. A positive correlation has been noted between TMB and TNB. However, TNB is directly used for neoantigen evaluation and may be considered an improved biomarker for immunotherapy compared with TMB (23–25). High TNB was associated with durable progression-free survival (PFS) in patients with non-small cell lung cancer (NSCLC) treated with programmed death 1 (PD-1) inhibitors (26). In addition, TNB correlated with clinical benefit in patients with metastatic melanoma treated with cytotoxic T-lymphocyte-associated protein 4 (CTLA4) inhibitors (27). Similarly, a phase I/II trial performed in patients with stage IV melanoma demonstrated that their clinical benefit was associated with a proposed immune activation signatures score. Among the score items, high TMB and predicted TNB were significantly associated with improved PFS and overall survival (28). The present review investigated the application of TNB as a biomarker in immunotherapy and other therapies and provided an in-depth discussion of the mechanisms, clinical application and challenges of this biomarker.

## Neoantigen Prediction

In general, *in silico* analysis on genome sequencing can aid the selection of immunogenic neoantigen peptides. Neoantigen prediction is usually performed prior to selecting immunogenic neoantigens to reduce the burden of immunogenicity testing by decreasing the number of candidate peptides. This is a necessary step in developing personalized immunotherapy. Several important steps are involved in neoantigen selection, including intracellular processing and transportation, the stability and affinity of peptide-MHC complex binding, the diversity of T cell receptors (TCR) and the recognition by TCR. In addition, the difference between the prediction algorithms is also important. Neopepsee is a neoantigen prediction algorithm that automatically extracts mutated peptide sequences and expression levels, and combines multiple immunogenic features to construct a machine-learning classifier (29). The application of deep learning to the determination of large human leukocyte antigen (HLA) peptides and genomic data sets from various tumors can aid the development of a computational model for neoantigen prediction (30). An additional prediction algorithm can determine the priority of neoantigens and discover immune characteristics in cancer immunotherapy by the classification of human neoantigen/neopeptide data into three categories based on different mutation positions (anchor mutation, MHC-contacting position and TCR-contacting position) (31).

Several computational pipelines have been developed for neoantigen prediction. However, the majority of them are based on peptide affinity with MHC (32–34). Furthermore, neoantigen prediction can be performed by prioritizing predicted peptides based on mutant allele expression, mutation clonality, MHC presentation, and T cell recognition, either alone or in combination (35–38). A Cauchy-Schwarz index of neoantigens score was proposed and the effects of both clonality and MHC binding affinity were included in order to accurately determine the concentration of neoantigens in truncal mutations (39). An additional prediction model was developed by integrating peptide presentation and recognition into antigenic determinant immunogenicity *via* the use of specific parameters.

To establish a global neoantigen prediction algorithm standard, several institutions established the tumor epitope selection alliance, which is a bioinformatics consortium with scientists from well-respected neoantigen research groups. These institutions independently mine the open database of tumor sequencing, predict potential neoantigens and rank candidate peptides. Different predictions may be collected and cross-matched to reach a final optimized consensus. This integration incorporates aspects of binding affinity, tumor abundance, stability and peptide identification in addition to antigen presentation. Therefore, higher precision would be expected (40).

## The Mechanisms of TNB Formation

Any form of genomic instability, including single -nucleotide variation (SNV), frameshift mutations, splicing variations or chromosome rearrangement, may result in TNB. The genomic instability can result from abnormalities in either DNA replication or mismatch repair (MMR) (41). The high-fidelity process of DNA replication requires replicative DNA polymerases, exonucleolytic proofreading and MMR. Abnormalities that may occur in any of these parts contribute to genetic instability. Inactivation of DNA polymerase leads to excessive mutations, such as ultra-hypermutated phenotype. Defective MMR (dMMR) leads to microsatellite instability (MSI), which is an ultra-hypervariable phenotype of short repetitive DNA sequences and SNV. Following exposure to either exogenous (smoking, ultraviolet radiation, chemicals, ionizing radiation) or endogenous (reactive oxygen species, endocrine abnormalities) mutagens, dMMR/MSI facilitates carcinogenicity and paradoxically increases TNB, which in turn enhances immunogenicity (42–46).

Although TNB is a biomarker of immunotherapy, current knowledge regarding its function is limited. Primarily, TNB analysis was performed on SNV (47). However, other genetic aberrations may produce comparable or even more immunogenic neoantigens (23). For example, neoantigens were found from a data set of gene fusion-positive tumors (48). Splice variants are also sources of neoantigens. High expression of PD-1 and programmed death-ligand 1 (PD-L1) were observed in tumors with splice variants (49). In addition, a new class of neoantigens was discovered, which was derived from intra- or inter-chromosomal rearrangements (50).

Various studies have analyzed the frequency of specific somatic mutations in multiple types of cancers, which demonstrated that the frequency of non-synonymous mutations varied greatly, ranging from ~0.001/Mb to higher than 400/Mb. The mutation frequency was very prominent in melanoma and lung cancer (20, 51). Notably, Samra Turajlic et al. analyzed and compared the counts of Insertion-and-deletion-derived tumor-specific neoantigens in pan-cancer, both SNV-derived neoantigens and frameshift indel-derived neoantigens in the study showed that melanoma, lung cancer, bladder cancer, colon cancer, and head and neck cancer with high TNB (47). Mutations in lung cancers can be attributed to direct DNA damage from cigarette smoke carcinogens. A significant dose-response association of smoking history with genetic alterations has been noted in advanced non-small-cell lung adenocarcinoma with regards to cancer-associated pathways and their corresponding mutant antigens (52, 53). Ultraviolet stimulation is the main factor leading to high TNB in melanoma (54). In other common cancer types, such as colorectal or endometrial cancer, which harbor DNA polymerase epsilon mutations, increased TNB is attributed to endogenous mutations (55, 56). However, in certain tumors, such as bladder cancer, the mechanisms underlying the formation of TNB are complex, including the apolipoprotein B mRNA editing enzyme catalytic polypeptide family, smoking, viral infection and genetic fusions (57).

A previous study dissected the genetic heterogeneity during the evolution of a primary osteosarcoma tumor to its metastatic variant. Metastases exhibited higher TNB compared with primary tumors, possibly due to the accumulating mutations in DNA damage response genes (58). Different mutational landscapes exist between the primary and metastatic sites and in the subclones noted inside different regions of a tumor (intra-tumoral heterogeneity; ITH). The presence of ITH suggests that the tumor cannot elicit equal immunity. Patients with tumors exhibiting high TNB and low ITH are more likely to benefit from immunotherapy (59, 60).

## TNB Correlates With Tumor-Infiltrating Lymphocytes

**Figure 1:**
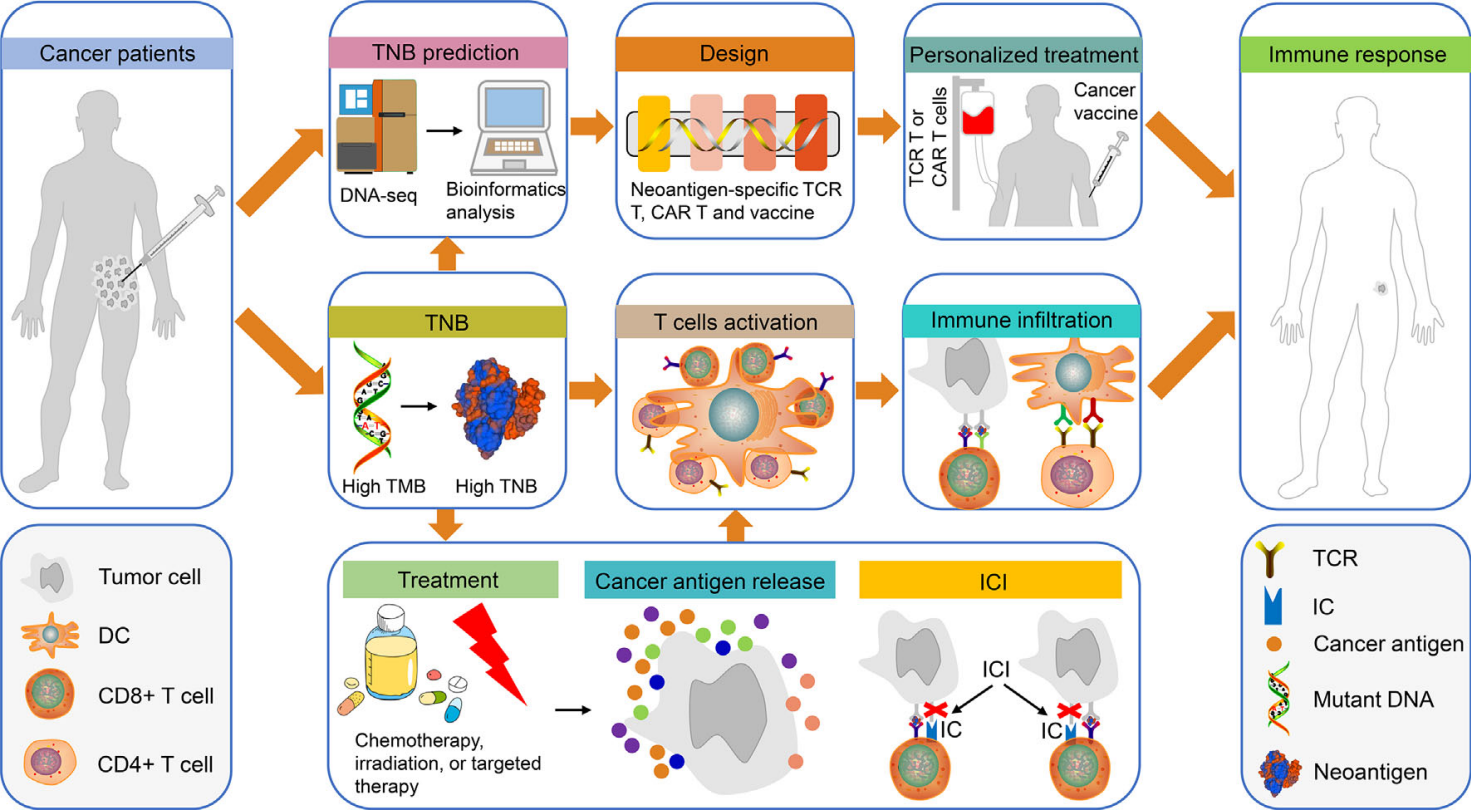
The use of TNB in personalized immunotherapy for patients with cancer. Tumor tissues from patients with cancer were obtained for DNA sequencing and bioinformatics prediction of TNB. Subsequently, the TCR-T, CAR-T and the cancer vaccines were designed based on TNB for personalized treatment. Finally, the patients exhibited an immune response to cancer cells. In addition, high TMB in patients with cancer can produce high TNB, which will promote T cell activation and immune cell infiltration, thereby causing an immune response in these patients. It is interesting to note that chemotherapy, radiotherapy or targeted therapy can promote the release of cancer antigens in patients with high TNB, which in turn can enhance the therapeutic effects of ICI-based treatment. This type of treatment will activate T cells, increase immune infiltration and produce immune responses. TNB, tumor neoantigen burden; TCR-T, neoantigen-specific T cell receptor engineered-T cell; CAR-T, chimeric antigen receptor-T cell; ICI, immune checkpoint inhibitor.

Neoantigens alone are not sufficient to mount an effective immune response and tumor-infiltrating lymphocytes (TILs) are also required for this process (61, 62). High TNB can promote the recognition and activation of T cells, which in turn increase TILs and improve the immune response of cancer patients to cancer cells (**Figure 1**). Colorectal tumors with dMMR exhibit neoantigen-stimulated lymphocyte infiltration and increased levels of inflammatory cytokines. In the absence of high TMB, high TNB alone correlates with the inflammatory microenvironment (63). Moreover, lung adenocarcinoma patients with high TMB presented with enhanced infiltration of activated CD4^+^ and CD8^+^ T cells, while the mutations detected could accurately predict the increased TNB and T cell infiltration. In addition, TNB was significantly associated with the expression levels of M1 polarized macrophage genes, namely PD-1, PD-L1, interferon-γ (IFNγ), Granzyme B FAS ligand and other immune-associated genes (64). The correlation between TNB and TILs has been verified in multiple studies (64–66).

High TNB correlates with the abundance of TCR clonality and the infiltration of activated CD4^+^ and CD8^+^ T cells (50, 67). This may be mediated by the elevated expression of chemokines induced by IFN-γ, such as chemokine (C-X-C motif) ligand (CXCL) 9, and by the recruitment of T cells or myeloid dendritic cells (45, 68, 69). In the majority of the cases, high TNB can predict inflammatory microenvironment and optimal immune response. The infiltration of different immune cell subgroups is commonly noted in a special spatial compartment termed tertiary lymphoid structure (TLS). The mechanism by which TLS responds to the tumor microenvironment is actively studied. A previous report indicated that transforming growth factor β1 induced co-expression of CXCL13 and CD103 in CD8^+^ T cells, providing a potential link between CD8^+^ T cell activation and B cell migration (70, 71).

## The Role of TNB in Tumor Immunotherapy and Other Therapies

### Tumor Vaccine and T Cell Therapy

The use of individualized neoantigen vaccines and neoantigen-specific T cell therapy is actively explored. This topic has been well described in previous review articles (72–76) and will not be covered in the present review. It should be noted that individualized vaccines against a single neoantigen demonstrated limited efficacy. The use of a complete tumor lysate vaccine or a personalized vaccine containing multiple neoantigens can improve patient outcomes (6, 77). The dendritic cells were pulsed with oxidized autologous whole-tumor cell lysate, which was proved as an effective vaccine in patients with ovarian cancer. This vaccine amplified T cell responses against recognized neoepitopes and elicited *de novo* responses for previously unrecognized neoepitopes (78). An additional study tested the efficacy of an adenoviral vaccine consisting of multiple neoantigens. This vaccine facilitated T cell infiltration and expanded the breadth and efficacy of the TCR repertoire following ICI treatment (79). Tumor cell lysates differ in their efficiency as vaccines. A direct comparison of 2 autologous tumor cell lysate (with different TMB) vaccines demonstrated that the lysates with lower TMB inhibited tumor growth more efficiently. Thus, it may be considered that the neoantigen quality outranked the quantity (80).

### Radiotherapy and Chemotherapy

Radiotherapy or chemotherapy can facilitate immunotherapy and is possibly attributed to increased exposure of neoantigens (81) (**Figure 1**). In addition to direct tumoricidal effects, radiotherapy converts the irradiated tumor cells into an *in situ* vaccine (82, 83). In locally advanced rectal cancer, neoadjuvant chemoradiotherapy induced new neoantigen epitopes and altered the immune function of the hosts (84). Similarly, patients with relapsed anal squamous cell carcinoma exhibited high TNB following radiochemotherapy and indicated objective responses to PD-1 inhibitors (85). In bladder cancer, dual poly (ADP-ribose) polymerase and PD/PD-L1 inhibition is used to improve disease prognosis (86). The standard treatment for high-grade serous ovarian carcinoma is surgery and/or chemotherapy. However, only dismal results are obtained, whereas in a subgroup of patients harboring high TNB, an improved prognosis was achieved. Moreover, an additional study demonstrated that TNB could be used to determine the prognosis of patients with clear cell renal cell carcinoma who received either surgery alone or surgery combined with adjuvant therapies (87–89).

The more important aspect is that pediatric tumors exhibit low TMB at diagnosis, whereas the levels of this biomarker increase when the tumor is exposed to chemoradiotherapy, resulting in neoantigen targets (90, 91). The majority of the pediatric tumors have less TIL and low MHC expression. In addition, the immune system of the children is immature. Consequently, the current immunotherapy alone is not sufficient to treat pediatric tumors efficiently. The combination of immunotherapy with conventional radio- and chemotherapy can achieve an improved survival benefit (92, 93).

### TNB and Responses to ICIs

High TNB produces neoantigens, contributing to an inflammatory microenvironment, which ultimately leads to improved outcomes following ICI therapy (**Figure 1**). A previous study performed in patients with NSCLC, who exhibited high TMB or genetic defects in the DNA repair pathway, demonstrated that they benefited from ICI treatment. At least in one responder, neoantigen-specific CD8^+^ T cell responses paralleled with tumor regression (26). It has also been shown that patients with melanoma, who are treated with CTLA4 inhibitors, demonstrated a significant association of TMB, TNB and cytolytic marker expression with clinical benefit (27). Recently, a model of immunotherapy score (ITS) mutation was proposed for predicting the response of patients with melanoma to ICI treatment. Patients with high TMB and TNB exhibited higher ITS scores and immunotherapy sensitive features (94).

A scoring system based on neoantigen concentration combined with clonality and MHC binding affinity predicted responses to ICIs and the prognosis of patients with melanoma, lung and kidney cancers (39). Neoantigen concentration levels were prognostic factors for patients with melanoma and chronic lymphocytic leukemia treated with ICIs (29). However, paradoxical results were reported, indicating inferior PFS with high TNB in patients with multiple myeloma. The possible explanation for these findings was that the disease progression caused reduced efficiency of T cell recruitment (24).

The diversity and clonality of neoantigen-responsive TCR are also potential biomarkers for immunotherapy (95, 96). Additional research studies in this topic will aid the successful application of immunotherapy.

## Challenges and Perspectives

The rapid development of immunology and bioinformatics has enabled the successful prediction of neoantigens. However, the standard pipeline for neoantigen prediction and the optimized cut-off value for TNB are unknown, since it is an emerging biomarker. In addition, the presence of specific mutations causing ITH should be taken into consideration (97). Heterogeneity exists not only locally but also between primary lesions and their successive metastases (98). Neoantigen prediction is currently hindered by the difficulty in exploring the entire tumor through a partial biopsy (99).

Several obstacles hinder the patient immune response, such as the loss of HLA (100–103). Failure of successful HLA presentation renders the candidate neoantigens ineffective *in vivo*. Therefore, TNB alone cannot accurately predict the immune response. Currently, the personalized detection of circulating tumor DNA is considered a powerful tool for the dynamic monitoring of TNB (16, 80, 104). However, the clinical application of TNB is still limited. Previous evidence has shown that the quality of neoantigens may be more important, since high-quality neoantigens can confer higher immunogenicity (105). According to our opinion, the real-time status of the high-quality neoantigen burden can monitor the treatment response more effectively. The construction of a neoantigen vaccine library and a neoantigen-responsive T cell receptor repertoire can provide a more comprehensive and personalized antitumor treatment. Future studies should focus on assessing the quality of TNB.

## Author Contributions

CW and PW conceptualized the study. PW wrote and edited the manuscript. YC helped revise the manuscript. All authors contributed to the article and approved the submitted version.

## Funding

This work was supported by Clinical Research Incubation Project, West China Hospital, Sichuan University (2020HXFH056).

## Conflict of Interest

The authors declare that the research was conducted in the absence of any commercial or financial relationships that could be construed as a potential conflict of interest.
